# Neural circuits controlling behavior and autonomic functions in medicinal leeches

**DOI:** 10.1186/2042-1001-1-13

**Published:** 2011-09-28

**Authors:** Damon G Lamb, Ronald L Calabrese

**Affiliations:** 1 Department of Biology, Emory University, 1510 Clifton Road, Atlanta, GA 30322, USA

**Keywords:** central pattern generation, behavioral decision, *Hirudo *sp., swimming, crawling, heartbeat, neuronal circuits

## Abstract

In the study of the neural circuits underlying behavior and autonomic functions, the stereotyped and accessible nervous system of medicinal leeches, *Hirudo *sp., has been particularly informative. These leeches express well-defined behaviors and autonomic movements which are amenable to investigation at the circuit and neuronal levels. In this review, we discuss some of the best understood of these movements and the circuits which underlie them, focusing on swimming, crawling and heartbeat. We also discuss the rudiments of decision-making: the selection between generally mutually exclusive behaviors at the neuronal level.

## Introduction

The study of the neuronal basis of movement, both behavioral and autonomic, is often stymied by an inability to move between cellular, network, system and behavioral levels and an inability to disambiguate the role of sensory feedback and centrally generated commands [1]. While many model systems are amenable to analysis at one or two levels, invertebrates often allow for analysis across all. The stereotypy of their relatively simple nervous systems allows for reliable identification of the same neuron between animals, and behaviors can often be evoked in semi-intact preparations that facilitate cellular and circuit level analyses [2]. Identifiable neurons allow for detailed study of circuits, constituent neurons, the neural patterns they produce and the resulting movements, both behavioral and autonomic. This ability to cross levels allows for the study of the rudiments of decision-making [3,4]. Moreover, it is usually easy to evoke fictive motor patterns in invertebrates, rendering feasible the study of the interaction between sensory feedback and centrally generated commands by combining observations in fictive preparations with those from semi-intact preparations [5]. Thus, we can study neurally controlled movement from the whole animal down to the contribution of individual neural properties and parse the role of sensory feedback from centrally generated commands. These attributes of invertebrate preparations, among others, have allowed for many significant contributions to our understanding of the brain and the neural bases of behavior and autonomic movement [6-8]. Leeches are a particularly suitable organism in which to study the neural bases of movement, and in this review we will focus on a subset of leech behaviors and autonomic movements for which the neural circuits have been intensively studied: swimming, crawling, heartbeat and decision-making.

When sufficiently motivated by sensory input that either suggests a potential meal or indicates a disturbance, leeches will initiate targeted locomotion in the form of swimming or crawling [9,10]. Gentle touch or other minor sensory input can elicit a variety of avoidance behaviors, depending on its location and its environmental and internal context, including local bending, shortening and whole-body shortening [10]. Leeches express other special behaviors, such as mating, but the underlying neural circuits have yet to be elucidated because of the difficulty in eliciting them, although the induction of fictive mating behavior recently became possible [11]. In addition to overt behaviors, the leech circulatory system is continually pumping blood. A leech's bilateral heart tubes require constant excitatory drive from motor neurons to produce the complicated motor pattern. We will discuss these behaviors, the neural circuits that generate them and decisions between competing behaviors.

### Swimming

**Figure 1:**
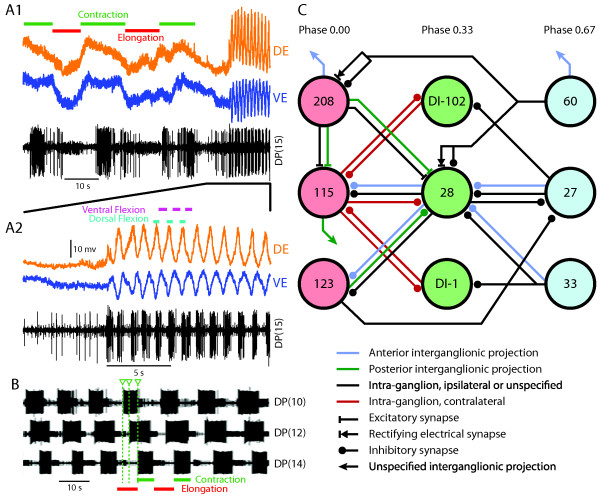
**(A1) Voltage-sensitive dye recording of dorsal and a ventral excitatory longitudinal motor neurons, as well as a nerve, on which dorsal excitatory motor neuron bursts are recorded, in midbody ganglion 15**. (Data in Figure 1A were kindly provided by Kevin Briggman from experiments described in [30].) Initially, in phase oscillations of the dorsal longitudinal excitatory (DE) and ventral longitudinal excitatory (VE) motor neurons with a period of about 20 seconds indicate fictive crawling. At the end of the recording, fictive swimming behavior commences. (A2) Zoom of fictive swimming motor pattern from (A1): DE and VE motor neurons oscillate out of phase and with a period of about one second. (B) Dorsal posterior (DP) nerve recordings from multiple ganglia during crawling demonstrate the phase lag between ganglia from front to rear. Downward arrows and lines indicate the start of a motor neuron burst for a selected cycle of fictive crawling. (Data kindly provided by Karen Mesce and Joshua Puhl.) (C) Simplified circuit schematic of a segmental oscillator of the swimming CPG and its intersegmental connectivity: component neurons are broken down into three phase groups, 0, 0.33 and 0.67, with the inter- and intrasegmental connectivity indicated. Less important elements are omitted from the schematic, that is, cells VI-2 and VI-119. The anterior projections are replications of the intrasegmental connectivity, whereas the posterior projections differ. Inhibitory motor neurons DI-102 and DI-1 participate in and can strongly influence the pattern produced. Only cells 28 and 27 have strictly reciprocal connectivity. (Original artwork adapted from [12], Figure 10, and from [5], Figure 15.)

Leeches swim with a dorsoventral, approximately sinusoidal, undulatory traveling wave with a wavelength of approximately one body length [12]. Swimming begins with undulations at the anterior of the leech that travel toward the posterior sucker. Upon initiation of swimming, dorsoventral flattener muscles contract and flatten the entire leech, which takes on a body form reminiscent of a ribbon with a flared posterior sucker paddle. Dorsal and ventral longitudinal muscles are primarily responsible for swimming undulations and are innervated by dorsal excitatory motor neurons (DE-3, DE-5, DE-18 and DE-107), dorsal inhibitory motor neurons (DI-1 and DI-102), ventral excitatory motor neurons (VE-4, VE-8 and VE-108) and ventral inhibitory motor neurons (VI-2, VI-7 and VI-119) [10,13-15]. Alternating contraction and relaxation of the dorsal and ventral muscles results in rhythmic bending of the body segments with a period of 0.3 to 1.0 second and a phase lag, or intersegmental delay normalized to period, of 0.044 to 0.1 second per segment, which generates the traveling wave that is leech swimming [10,16]. In response to various inputs, isolated or semi-intact preparations can exhibit fictive swimming, in which DE and VE motor neurons show alternating bursts of activity within a period range similar to that of swimming (Figure 1A2) and intersegmental coordination with front-to-rear phase lags.

### Initiation

Swimming can be experimentally elicited, either as an escape mechanism or for directed locomotion. A moderate touch or a more significant, higher-intensity contact can initiate swimming in sufficiently deep water. When the leech is hungry, either pressure or light-dark waves in water will elicit from the leech targeted locomotion toward the apparent source [9,17]. Alternatively, activation of various sensory neurons, trigger neurons and command neurons can elicit fictive swimming in the isolated nerve cord. The sensory stimuli that activate swimming are transduced by several classes of sensory neurons, including the sensillar movement receptors (touch-sensitive, pressure-sensitive and nociceptive), in addition to those of the light-sensitive organs. Stimulation of sensory neurons results in the activation of trigger and command neurons through either direct or polysynaptic connections [10,16,18]. Trigger and command neurons are located predominantly in the head brain, although a recently identified cell, E21, has functions similarly to the trigger cell 1 (Tr1) neuron and is located in the rearmost midbody ganglion [19]. The downstream targets of these trigger and command neurons include cells 204 and 205, which are found in midbody ganglia 9 through 16 [10,16]. These cells function as "gating" command neurons, as their activity initiates and maintains swimming behavior [16,20,21].

### The circuit

The central pattern generator (CPG) circuit that produces the swimming motor pattern in leeches is composed of complex segmental oscillators which rely heavily on intersegmental connectivity to generate a robust motor pattern. Midbody ganglia contain a bilateral, triphasic oscillator composed predominantly of bilaterally paired interneurons with significant interganglion connectivity (Figure 1C). The constituent neurons of this oscillator can be grouped by the relative timing of their bursts of activity into phase 0 (cells VI-2, 115, VI-119 and VI-123 as well as the unpaired cell 208), phase 0.33 (cells DI-102, DI-28 and DI-1) and phase 0.67 (cells 60, 27 and 33) [16]. These weak segmental oscillators can be accurately modeled by a recurrent cyclical inhibition network with three members [1], although the activity in the actual network arises from a more complicated interaction of inhibition and excitation; the constituent neurons are not thought to be intrinsic bursters. Furthermore, few members of the oscillator have reciprocal inhibitory connectivity.

Neurons of the CPG receive indirect input from sensory neurons, as well as direct input from trigger, command and gating neurons and synapse onto both inhibitory and excitatory motor neurons. Furthermore, there are significant asymmetric connections between the swim oscillators in neighboring ganglia that help maintain an intersegmental phase lag along the longitudinal (front-to-rear) axis of the animal (Figure 1C), although the sensory input from the stretch receptors also plays a key role in producing the appropriate phase lags [22,23]. In addition to local projections, touch and pressure sensory neurons directly project to several interneurons in the head brain, such as trigger neurons and the E21 neuron, that play decision-making and initiation roles.

Although an isolated nerve cord can express a fictive swimming motor pattern, some characteristics of the pattern in the intact animal are altered by sensory feedback. In particular, intersegmental coordination is affected by the stretch receptors in the longitudinal muscles [22,23]. Certain ganglia can express a rudimentary fictive swim pattern when isolated; however, this pattern is not robust and terminates quickly. As such, they are considered weak, independent segmental oscillators, and intersegmental connectivity is critical in establishing a robust swimming pattern. Furthermore, as the number of ganglia in an isolated nerve cord is reduced, the intersegmental phase lag increases, reinforcing the importance of intersegmental connectivity in establishing the correct pattern. The ventral stretch receptors (VSRs) have an electrical connection to cell 33 and polysynaptic connections to cells 28, 115 and 208 [24], all of which are components of the segmental oscillator network. Dorsal stretch receptors have also been identified, but have not been studied in as much detail. Without the sensory feedback, the period of the swimming motor pattern is longer and the phase lags are shorter, although the resulting pattern in intact animals is a balance between the intrinsic periods and lags of the isolated cord due to sensory feedback [25-27]. Furthermore, stimulation of the VSRs can entrain the swimming rhythm, suggesting that such ongoing sensory feedback allows for continual adaptation of the pattern to the fluid dynamics which occur during swimming [23,28].

### Crawling

The second primary mode of locomotion that leeches exhibit is crawling in various forms. In the best studied form, the leech plants its posterior sucker, extends its body with a wave of circular muscle contraction from anterior to posterior, then plants its anterior sucker, releases the posterior sucker, shortens its body with a front-to-rear wave (this time by longitudinal muscle contraction) and finally anchors its posterior sucker. To achieve this elongation and shortening, the circular muscles and longitudinal muscles within each segment contract in antiphase. In air, each cycle or "step" typically moves a leech two-thirds to three-fourths of its length, and it typically takes 3 to 10 seconds in intact animals [29-31]. As with swimming, fictive crawling can be generated in isolated preparations (Figures 1A and 1B), but with a cycle duration as long as 20 seconds.

### Initiation

Many of the same sensory inputs that initiate swimming can initiate crawling. In addition to physical or electrophysiological stimulation, neuromodulators can initiate fictive crawling, even in the absence of the head brain or tail brain, which are otherwise required [30]. Dopamine elicits fictive crawling in isolated preparation, even individual ganglia [32]. This observation supports the ideas that each ganglion contains a crawling unit burst generator and that these coordinate with one another to produce the complete crawling motor pattern [32,33]. As the composition of the crawling CPG appears to support the theory of unit burst generators as a fundamental component of motor pattern generation, the details of the responsible circuits are ripe for further inquiry.

### The circuit

The crawling CPG is less well understood than other behavioral circuits in leeches. The relative activity patterns of many motor neurons involved in crawling have been described [34] and several command neurons have been identified [18,33], but the constituent neurons of the crawling unit burst generator apparently present in each midbody ganglion have not yet been specifically identified, although many candidates with correlated activity have been [3,30]. Consistent with the intact motor pattern, in an isolated preparation, the motor neurons within each ganglion are rhythmically active in two groups. The motor neurons responsible for the contraction phase, that is, the DE, VE and annulus erector motor neurons, exhibit bursts of activity in antiphase with the motor neurons responsible for elongation, including circular muscle motor neurons (CV) and longitudinal muscle inhibitory (VI and DI) motor neurons [3,32,33]. Furthermore, intersegmental delays in the fictive pattern show a front-to-rear progression and period in the range of crawling (Figure 1B). There appears to be a great deal of overlap between the swimming and crawling CPGs [3], although the nature of the connectivity within and between the two circuits has yet to be elucidated. What is known is that (1) the segmental crawl unit burst generators project to neighboring ganglia and influence the pattern produced and (2) the rearward projections go farther and have more significant influence [33].

### Decision-making in the leech

Leeches constantly make choices about how to respond to external stimuli and internal drives (such as hunger). It is these behavioral choices that we consider decision-making, despite the simplicity of the leech's nervous system. No matter how complex, a decision may be broken down into elemental choices, and the role of context in the form of sensory environment, internal state and experience influences each of these choices [4]. We next discuss three behavioral choices relevant to swimming and crawling and what is known of their neuronal circuitry and context dependence.

### Swim or shorten

When a leech is touched at its front end, it reliably shortens, even if it was swimming at the time. Swimming and shortening are incompatible behaviors, and although the circuitry for shortening is not well-understood, it is possible to determine how swimming is inhibited once the "decision" to shorten has been made. The most powerful command neuron for swimming, cell 204, is strongly inhibited by stimuli that elicit shortening, but two swim trigger neurons, Tr1 and swim exciter 1 (SE1), are excited. These observations in turn suggest that cell 204 is dedicated to swimming but that the trigger neurons are multifunctional.

### Swim or crawl

Whereas tactile stimuli at the front end of the leech elicit shortening, stimuli at its rear generally elicit either crawling or swimming. How is the decision made between swimming and crawling? Water level has something to do with it, because leeches in deep water tend to swim, whereas those partially submerged or in shallow water tend to crawl [18]. Briggman *et al*. [3] investigated this decision in the isolated nerve cord by simultaneously recording the membrane voltage of almost all the neurons on the ventral surface of a segmental ganglion using voltage-sensitive dyes. By focusing on a midbody ganglion and stimulating a nerve electrically, they could evoke swimming or crawling with roughly equal likelihood. They discovered a small set of neurons with covarying activity prior to initiation of either motor pattern that discriminated swimming from crawling at an average of 290 milliseconds earlier than any later (individually) discriminating neurons. Cell 204 is one of the late discriminators, indicating that it is indeed a command neuron implementing the "decision" of the early discriminating group. One of the earlier discriminators is cell 208, and depolarizing this neuron biases the nerve cord to produce the crawl motor pattern, while hyperpolarizing this neuron biases this nerve cord toward the swimming motor program. Originally identified as a member of the swimming pattern generator, cell 208 was more recently found to be a member of both the swimming and the crawling networks: After apparently participating in favor of crawling during a swim-crawl decision, it participates in either the crawling network, if it wins, or the swimming network, if it loses

### Ignoring tactile input while feeding

Feeding in leeches suppresses all touch-mediated behaviors, including shortening, swimming and crawling. This decision is made by a generalized release of serotonin from as yet unidentified sources that presynaptically inhibit release at synapses from pressure-sensory neurons [20,35]. Although many candidate neurons, including the Retzius neurons, have been proposed, it is not yet clear which are specifically responsible [35]. This generalized sensory gating suppresses even the local bending avoidance reflex, which is compatible with feeding, unlike swimming, crawling and shortening. Thus, this mechanism is not compatible with more selective decision-making and clearly prioritizes feeding.

### The heartbeat neural control system

**Figure 2:**
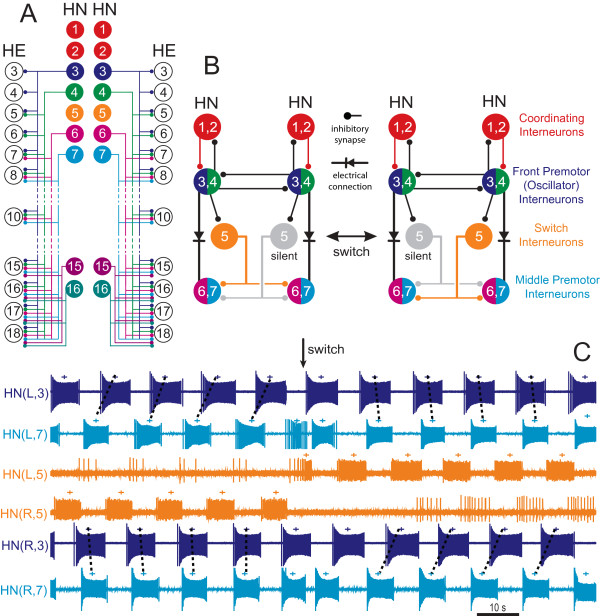
**The heartbeat control system of medicinal leeches: heart motor neurons and the heartbeat central pattern generator**. **(A) **Bilateral circuit diagram including all the identified heart interneurons (HNs) of the central pattern generator (CPG) showing the inhibitory connections from the HNs of the leech heartbeat CPG onto heart (HE) motor neurons. **(B) **Circuit diagram of the identified HNs of the core CPG showing their synaptic interconnections. The two possible states of the heartbeat CPG are illustrated, one with the left switch interneuron quiescent and the right switch interneuron active (corresponding to left synchronous), and the other with the left switch interneuron active and the right switch interneuron quiescent (corresponding to left peristaltic). In **(A) **and **(B)**, large filled circles represent cell bodies and associated input processes. Lines indicate cell processes, small filled circles indicate inhibitory chemical synapses and diode symbols indicate electrical connections. HNs that have similar input and output connections are lumped together for ease of presentation. **(C) **Simultaneous recordings of a bilateral pair of front premotor interneurons (HN(3)), a bilateral pair of middle premotor interneurons (HN(7)) and the bilateral pair of switch interneurons (HN(5)) during a switch in coordination mode from left synchronous to left peristaltic as indicated in the circuit diagrams in **(B)**. Body side indicated by R or L in the HN index.

Heartbeat is an autonomic movement in vertebrates, but in medicinal leeches it is more analogous to vertebrate breathing than heartbeat, because it is a continuous, rhythmic motor pattern under direct neural control [10,36,37]. Rhythmic muscular constrictions of the two lateral heart tubes that run the length of the animal pump blood through the closed circulatory system. The heart tubes beat in a complex pattern that is not fully understood. While one heart tube peristaltically beats in a rear-to-front progression from midbody segment 15, developing a high systolic pressure and moving blood forward, the other heart tube beats nearly synchronously forward of segment 15, developing low systolic pressure and supporting blood flow rearward and into the periphery. Every 20 to 40 beats the two hearts reciprocally switch coordination states. The hearts are innervated in each segment by a bilateral pair of heart exciter (HE) motor neurons found in the third through eighteenth midbody segmental ganglia (HE(3) through HE(18)) (Figure 2A). The HEs are rhythmically active, and the segmental HEs' coordinated activity pattern determines the constriction pattern of the hearts. The same coordination modes, peristaltic and synchronous, observed in the hearts occur in the HEs. On one side they are active in a rear-to-front progression, and on the other they are active nearly synchronously forward of segment 15. The coordination of the motor neurons along the two sides switches approximately every 20 to 40 heartbeat cycles. The rhythmic activity pattern of the HEs is a direct result of the cyclic inhibition that they receive from the heartbeat central pattern generator (CPG). The CPG comprises nine bilateral pairs of identified heart interneurons (HNs) that occur in the first seven ganglia, HN(1) through HN(7), and ganglia 15 and 16, HN(15) and HN(16) [10,38] (Figures 2A and 2B). HNs make inhibitory synapses onto HE motor neurons and among themselves. In addition, certain HNs are electrically coupled.

### The half-center oscillators

HN(1) through HN(4) constitute a core network that sets beat timing throughout the heartbeat CPG (Figure 2B), and the remaining five pairs of HNs are followers of these front pairs. There are two independent oscillators in the beat timing network: Each of the HN(3) and HN(4) bilateral pairs form a half-center oscillator based on strong reciprocal inhibitory synapses (Figure 2B). Synaptic and several intrinsic currents contribute to the oscillatory activity of oscillator interneurons [10,39,40]. These include a fast Na current that mediates spikes, two low-threshold Ca currents (one rapidly inactivating, *I*_CaF_, and one slowly inactivating, *I*_CaS_), three outward currents (a fast transient K current, *I*_A_, and two delayed rectifier-like K currents, one inactivating, *I*_K1_, and one persistent, *I*_K2_), a hyperpolarization-activated inward current (*I*_h_, a mixed Na-K current with a reversal potential of -20 mV) and a low-threshold persistent Na current (*I*_P_). The inhibition between oscillator interneurons consists of both spike-mediated and graded components, yielding oscillation in each HN half-center oscillator that is a subtle mix of escape and release [41]. Escape from inhibition is due to the slow activation of *I*_h _in the inhibited oscillator interneuron. Release from inhibition results from a waning of the depolarization in the active oscillator interneuron due to the slow inactivation of its *I*_CaS_, which slows its spike rate and thereby reduces its spike-mediated inhibition of the contralateral oscillator interneuron.

### Coordination in the beat-timing network

HN(1) and HN(2) act as coordinating interneurons that couple the two half-center oscillators [42-46]. HN(1) and HN(2) do not initiate spikes in their own ganglion; instead they have two spike-initiating zones, one each in midbody ganglia 3 and 4. Normally, the majority (> 85%) of spikes in the coordinating neurons are initiated in ganglion 4. The coupling between the two half-center oscillators causes the HN(3) and HN(4) oscillators on the same side to be active roughly in phase, although a small phase lead by the HN(4) oscillator is important for proper HE coordination. The mechanisms of coordination within the timing networks are consistent with interaction between two independent half-center oscillators that mutually entrain one another and assume the period of the faster oscillator, which then leads in phase.

### Control of motor neurons by heart interneurons

Six pairs of HNs are premotor, making ipsilateral inhibitory connections with a subset of the motor neurons in the network. These premotor interneurons are broken into front or oscillator premotor interneurons (HN(3) and HN(4)), middle premotor interneurons (HN(6) and HN(7)) and rear premotor interneurons (HN(15) and HN(16)) (Figure 2A).

### Heartbeat motor pattern switching

Switching between the peristaltic and synchronous modes (Figure 2C) is accomplished by the pair of HN(5) switch interneurons that link the front and middle premotor interneurons (Figure 2B). HN(3) and HN(4) inhibit the switch HN and excite ipsilateral HN(6) and HN(7) through electrical coupling [10]. The HN(5) switch interneurons then bilaterally inhibit HN(6) and HN(7) (Figure 2B) [10]. Only one of the switch interneurons produces impulse bursts during any given heartbeat cycle. The other switch interneuron is quiescent, although it receives rhythmic inhibition from the beat-timing oscillator (Figures 2B and 2C) [47]. Within a period approximately 20 to 40 times longer than the period of the heartbeat cycle (six to ten seconds), the quiescent switch interneuron is activated and the previously active one is silenced (Figure 2C). There are no synaptic connections between the switch interneurons, even though spontaneous switches in the activity state are always reciprocal. In the quiescent state, switch interneurons have a persistent outward current that is not voltage-sensitive and reverses around -60 mV [47]. This current turns off in a switch to the active state by hyperpolarizing the cell below threshold. Thus, in its quiescent state, a switch interneuron is inhibited by a persistent leak current. This switching appears to be controlled by an unidentified independent timing network extrinsic to the switch neurons that imposes a tonic inhibitory leak alternately on one of the two switch interneurons at a time.

The switch interneurons determine which side is in the peristaltic vs. the synchronous coordination mode by variably linking the timing oscillator to HN(6) and HN(7). Because only one switch interneuron is active at any given time and because they make bilateral connections to the middle premotor interneurons, there is an asymmetry in the coordination of the HNs on the two sides. The HN(6) and HN(7) middle premotor interneurons lead the HN(3) and HN(4) front premotor interneurons in phase on the side of the quiescent switch interneuron (peristaltic coordination) (Figure 2C). The HN(6) and HN(7) premotor interneurons and the HN(3) and HN(4) premotor interneurons are active roughly in phase on the side of the active switch interneuron (synchronous coordination). The exact phase of each of the middle premotor interneurons is determined by the balance of inhibition from the switch interneuron and excitation from the front premotor interneurons. The observed switches in the HEs' coordination state reflect switches in the activity state of the switch interneurons (Figure 2C). By shifting the coordination of the front and middle premotor interneurons, a switch in the activity state of the two switch interneurons shifts the coordination of the HEs between peristaltic and synchronous.

The recently discovered HN(15) and HN(16) are clearly premotor and provide input to the rearmost HEs (Figure 2A) [38]. Less is known about how they integrate within the CPG. They appear to receive electrical (excitatory) input from HN(6) and HN(7), and their phase changes with these inputs when they in turn are switched by the switch interneurons [48].

The heartbeat CPG can be conceptualized as two timing networks: a beat-timing network comprising the first four pairs of HNs (two oscillator pairs and two coordinating pairs) and an unidentified switch-timing network which governs the activity of the switch interneurons. The two timing networks converge on the switch interneurons, and, together with the HN(6), HN(7), HN(15) and HN(16) HNs, they make up the heartbeat CPG. The output of the CPG is configured into two coordination states of the HEs by the alternating activity states of the two switch interneurons.

## Conclusions

The medicinal leech is a fantastic organism in which to study the neural systems and circuits underlying behavior and autonomic movement. Even with our rich collective understanding of rhythmic motor pattern generation garnered from these and other animals, we have a great deal more yet to learn. Uncovering the details of centrally generated neural patterns and how they specifically interact with sensory feedback, and with one another, to produce adaptable, behaviorally meaningful motor patterns is an important goal of research in the medicinal leech. For example, a more complete picture of the circuit responsible for crawling is within reach, as is a better understanding of the contribution of individual neural properties of the constituent neurons of all of these circuits. Moreover, we are just beginning to exploit the full potential of the leech for the investigation of behavioral choice as well as the context dependence of these choices. We hope that this review provides readers with an appreciation of the depth of investigation leeches afford, as well as motivation and a foundation for future study.

## Abbreviations

CPG: central pattern generator; HE: heart exciter motor neuron; HN: heart interneurons; DE: dorsal longitudinal excitatory motor neuron; DI: dorsal longitudinal inhibitory motor neuron; VE: ventral longitudinal excitatory motor neuron; VI: ventral longitudinal inhibitory motor neuron.

## Competing interests

The authors declare that they have no competing interests.

## Authors' contributions

DGL drafted the introduction, swimming, crawling, conclusion and abstract sections. RLC drafted the decision-making and heartbeat sections. Both authors reviewed and revised the entire manuscript, and both read and approved the final manuscript.
